# The effect of experiences of fairness on honest behavior: a behavioral and neural study

**DOI:** 10.3389/fnbeh.2023.1279176

**Published:** 2024-01-08

**Authors:** Chen Zhang, Ming Yin, Jixia Wu

**Affiliations:** ^1^School of Educational Sciences, Xuzhou University of Technology, Xuzhou, China; ^2^School of Education, Soochow University, Suzhou, China; ^3^Jiangsu Province Engineering Research Center of Microexpression Intelligent Sensing and Security Prevention and Control, Nanjing, China

**Keywords:** fairness, altruism, honest behaviors, event related potentials, N2 component, P3 component

## Abstract

Prior studies have investigated the relationship between fairness and honesty. However, the differences in the focus of these studies have rendered cross-comparisons between them challenging and of limited value. Thus, this study explored how fairness impacts honest decision-making, focusing specifically on the effect of individuals’ experiences of fairness on their honest behavior. Experiment 1 explored the influence of different experiences of fairness on honest behavior in an altruistic context. In Experiment 2, we measured event-related potentials to further demonstrate the brain mechanisms of these experiences on altruistic dishonest behavior. In Experiment 1, we found that the reaction time for dishonest behavior was shorter for individuals who had positive unfairness experiences with high altruistic objects compared to low altruistic objects. Individuals who had negative unfairness experiences had shorter reaction times when engaging in dishonest behaviors for the sake of high altruistic objects compared to those with equitable experiences. In Experiment 2, in which there was an opportunity to lie for a highly altruistic object, those with fair experiences had greater N2 volatility and smaller P3 volatility than those with positive unfairness experiences. These findings highlight the value of integrating moral psychology and behavioral economics. Discriminant validity across fairness experiences can help illuminate the different motivations behind moral decisions.

## 1 Introduction

Most existing studies have examined how fairness impacts honesty within an egoistic context, with less emphasis on understanding how this relationship works in an altruistic context. Past investigations into the correlation between fairness and honesty (Gino and Pierce, 2009, 2010; Houser et al., 2012; Galeotti et al., 2017; Leib et al., 2019) reveal considerable disparities in measurement methodologies and specific research focus, rendering comparisons between distinct studies complex and of minimal referential value. Regarding measurement methodologies, the metrics for honest behavior are not consistent with the beneficiaries of lying, which fall into three categories: those that are purely self-interested (Houser et al., 2012; Galeotti et al., 2017), those that are purely altruistic (Gino and Pierce, 2009, 2010; Leib et al., 2019), and those involving both self and others (Gino and Pierce, 2009; Galeotti et al., 2017). It is important to note that the “others” in these studies were constant partners in fairness tasks. Thus, it is worth investigating whether individuals would adjust to preserve a balance between their personal interests and those of their partners when the balance of fairness is tipped. In terms of research content, researchers have selected varying facets of fairness variables as their breakthrough point, including objective fairness distribution events or fairness norms (Gino and Pierce, 2009, 2010; Galeotti et al., 2017), and subjective fairness perceptions (Houser et al., 2012; Leib et al., 2019). Compared to the wide range of fairness perceptions, fairness events are easier for individuals to recognize in daily life and to control in an experimental environment. Therefore, this study primarily explores how objective fairness events impact honest decision-making, focusing specifically on the effect of individuals’ experiences of fairness on their honest behaviors.

## 2 Literature review

### 2.1 Theoretical framework

According to equity theory (Adams, 1965), individuals evaluate the fairness of their own situation by comparing their own return on investment to that of their peers or partners. Individuals may suffer because of unfair treatment or events during this comparison, and they may hope to create opportunities that will change their return on investment or choose to discontinue certain tasks to relieve their suffering.

According to the cross-norm inhibition effect (Keizer et al., 2008), as a certain norm-violating behavior becomes more common, conformity to subsequent rules or norms deteriorates. Individuals may commit more honest norm violations in subsequent tasks if a fair norm or rule is broken.

#### 2.1.1 The effect of experiences of fairness on altruistic dishonest behaviors

Previous research has focused on the relationship between fairness and altruistic dishonest behavior (Gino and Pierce, 2009, 2010; Leib et al., 2019). Gino and Pierce (2009) conducted an experiment in which participants engaged in a lottery followed by an anagram task. Based on the initial amounts held by graders and solvers (USD 20), the participants were randomly divided into three groups: fairness, negative unfairness, and positive unfairness. In this task, a solver received two dollars for each correctly completed word anagram that was graded by a grader. Individuals who had experienced unfair allocations were more likely to lie to help or hurt their peers (i.e., other solvers) to maintain their fair allocations. Interestingly, Gino and Pierce (2010) further discovered that experiences of unfairness from random draws and judgments of others led to graders displaying immorally helpful or harmful behaviors.

Leib et al. (2019) focused on the emotional mechanisms that influence honest behavior when people perceive unfairness. They conducted a comparative study on helpful and harmful behaviors of three subgroups of participants who misrepresented their earnings. Using a dictator game (DG), the researchers added separate experimental groups, fair and unfair, and a control group. According to the findings, individuals in these three groups generally lied to help others. Only a small proportion engaged in dishonest and hurtful behavior, which was associated with elevated levels of anger and disappointment and low levels of gratitude.

Existing studies have also examined methodological and measurement inconsistencies. On the one hand, first, in terms of fairness independent variables, some studies focused on the impact of subjective perceptions of fairness on individual moral decision-making (Leib et al., 2019). Experiment 1 in Leib et al. (2019) analyzed subjective perceptions of unfairness by comparing perceptions of positive fairness with perceptions of negative unfairness.

Second, several studies focused on the effects of objective fairness distribution events on individual honesty (Gino and Pierce, 2009, 2010). In both of their studies, Gino and Pierce used a random draw to determine whether participants received a high initial amount or a low initial amount and determined the grouping of graders by comparing the amounts of graders to solvers. These groupings comprised fair treatment, disadvantageous unfair treatment, and advantageous unfair treatment. It is easy to see that the grader who is determined to withhold the solver’s payoff is the dictator in this game. However, this prior study differs from ours, which focused more on whether the recipient remains honest or chooses to be dishonest after experiencing a fair or unfair event.

On the other hand, in terms of the dependent variable indicators of honest behavior, although the objects of interest for dishonest behavior are not uniform and diverse, they are categorized into three main categories: those that are purely self-interested (Houser et al., 2012; Galeotti et al., 2017), that are purely altruistic (Gino and Pierce, 2009, 2010; Leib et al., 2019), and those that involve both self and others (Gino and Pierce, 2009; Galeotti et al., 2017). More significantly, the owners of interest in these studies were all playmates in the game task. When the fairness scales are shifted, it was worth investigating whether people make changes to maintain fairness in their own and their peers’ interests. However, our study aimed to find whether an individual will engage in dishonest behavior to help a third party unrelated to the game or task interaction after experiencing an unfair distribution.

Besides, while previous studies have primarily focused on adult participants, the participant population is somewhat ambiguous. Unlike previous studies (Gino and Pierce, 2009, 2010; Houser et al., 2012), recent studies (Galeotti et al., 2017; Leib et al., 2019) did not provide information about the demographic characteristics of the participants. To fill this void, our research concentrated on the impact of college students’ fairness experiences on their honest behavior.

As shown above, our study examined the influence of fairness experience on recipients’ altruistic dishonest decisions to help a third party in the dictator game. We recommend specifying the anticipated results in an altruistic context. In comparison to individuals with fair experiences, those who have had positive unfairness experiences may engage in more dishonest helping behaviors with shorter reaction times when dealing with a highly altruistic object. Conversely, individuals who have had negative unfairness experiences may exhibit fewer dishonest helping behaviors with longer reaction times. However, these results will not be observed when individuals are dealing with a low altruistic object. Specifically, we could not find any significant difference in dishonest helping behaviors and reaction time among diverse fair experiences groups.

#### 2.1.2 Neural evidence of the effect of fairness on moral behaviors

To offer a more general understanding of the effect of fairness experience on moral decisions, attempting to introduce brain dynamics to this effect is necessary. Individual behavior when making financial decisions is driven by fairness (Gino and Pierce, 2009), but it is ambiguous whether unfairness impairs pro-social decision-making and the corresponding brain processes. Previous research has not determined how specific patterns of activity in the brain appeared when multiple profiles of fairness influenced individuals’ moral decisions.

Previous studies proposed that certain brain dynamics could be linked to moral decisions (Xu et al., 2020; Miraghaie et al., 2022). Among studies that adopted the DG paradigm, Miraghaie et al. (2022) noted an unresolved question concerning whether specific patterns of brain activity correlate with different profiles of fairness. This question prompted the researchers to conduct an experiment that delved deeper into the topic. In their study, Miraghaie et al. (2022) used event-related potentials (ERPs) and randomly divided 39 participants into two groups (fair or selfish). All participants assumed the role of allocators in a DG. At the fronto-central cortical sites, the latency of ERP early negativity (N1) was 10 ms shorter in selfish DG players than in fair DG players. Subsequently, the positive wave (P2) in fair DG players suggested that more cognitive resources were required when they allocated the least gains to the other party. The selfish group’s P2 latency and amplitude supported the hypothesis that these participants tended to maximize their profit. In this study, participants in the ultimate game (UG) also assumed the role of responders. They discovered that, when selfish participants rejected less favorable endowment shares, medial frontal negativity (MFN) occurred earlier and with greater amplitude. In this case, all players received zero payoffs, demonstrating that MFN was associated with spiteful punishment in selfish participants. We discovered that the greater the selfishness, the greater the amplitude of the late positive component at posterior-parietal sites (LPC).

Analyzing the correlation between fair and moral behavior, Xu et al. (2020) showed that late positivity (LPP/P300) reflects the evaluation of the fairness of proposals and can predict subsequent pro-social decisions. They conducted two experiments to explore brain activity regarding the impact of unfairness on human charity gift behavior. In Experiment 1, participants acted as responders in a joint donation game, deciding whether to accept a donation proposal made by the proposer. In Experiment 2, the charity projects were classified as deserving or undeserving. In both experiments, the participants were more likely to reject an unfair donation proposal, showing that aversion to unfairness reduces pro-social motivation to assist an innocent third party, and the late positivity potential (LPP)/P300 elicited by fair offers was more positive than that elicited by moderately unfair and highly unfair offers.

However, there is limited research on the brain response to the relationship between an individual’s fair/unfair experience and their decision to be honest.

Building on previous studies, we conducted a study to test the hypothesis that individuals with positive unfair experiences, compared to those with fair experiences, would experience less conflict and use fewer cognitive resources in making dishonest altruistic choices when presented with a high altruistic object. This would result in smaller N2 and larger P3 amplitudes. Conversely, individuals with negative unfair experiences would experience greater conflict and allocate more cognitive resources to dishonest altruistic choices, resulting in larger N2 and smaller P3 amplitudes.

### 2.2 The present study

To examine the impact of individuals’ unfairness experiences on honest behaviors, we conducted two experiments to explore behavioral and brain responses to fair and unfair allocations in altruistic and charitable contexts. To investigate the manipulation of individuals’ fair/unfair experiences, we designed a DG in which dictators proposed three types of offers: If the participant (responder) accepted less or more than the proposer (dictator) suggested, they would experience negative or positive unfairness, respectively; otherwise, if the participant accepted the offer suggested by the proposer, they would experience fairness.

The first experiment explores the influence of different experiences of fairness on honest behaviors in an altruistic context. We proposed the following hypothesis:

*Hypothesis 1*: In an altruistic context, individuals who have had positive unfairness experiences with high altruistic objects may engage in more dishonest helping behaviors with shorter reaction times compared to individuals with fair experiences. Meanwhile, individuals who have had negative unfairness experiences may exhibit fewer dishonest helping behaviors with longer reaction times. However, these results may not be observed when individuals are dealing with a low altruistic object. Specifically, we did not find a significant difference in dishonest helping behaviors and reaction time among diverse fair experiences groups.

The second experiment further demonstrated the brain mechanisms behind experiences of fairness and their impact on altruistic dishonest behaviors. We aimed to replicate the results of Experiment 1. In addition, we hypothesized:

*Hypothesis 2*: Individuals with positive unfair experiences would show smaller N2 and larger P3 amplitudes compared to those with fair experiences. Conversely, individuals with negative unfair experiences would show larger N2 and smaller P3 amplitudes compared to those with fair experiences. These results, however, may not be observed in less altruistic subjects. Specifically, there would be no significant difference in N2 and P3 amplitudes among diverse fair experiences groups.

## 3 Experiment 1: Experiences of fairness and honest behavior

### 3.1 Materials and methods

#### 3.1.1 Participants

Two hundred and eighty-seven participants (171 females, 116 males; age range = 18–24) were recruited from three universities in East China. Two female participants were excluded due to duplicate numbers. Before the experiment, the participants signed an informed consent form. The experiment was approved by the Soochow University’s ethics committee, and participants were randomly assigned numbers to ensure anonymity. Participants were randomly assigned to one of three groups: fair experience, negative unfair experience, and positive unfair experience. The participants were compensated for their participation. Participants received a small monetary reward after completing the experiment.

#### 3.1.2 Design

##### 3.1.2.1 Allocation of experiences of fairness

This was a virtual experiment scenario. Participants jointly distributed a 400 Yuan payoff after completing a typing task with a partner. The participants then drew roles, becoming either the allocator (who has the power to decide how to allocate the payoff) or the recipient (who can only accept the allocation plan chosen by the allocator). In practice, the participant can only play the recipient role. When the recipient received 100 Yuan (at which point the allocator receives 300 Yuan), they entered the negative unfairness group; when the recipient received 200 Yuan, they entered the fairness group; and upon receiving 300 Yuan, they entered the positive unfairness group.

##### 3.1.2.2 Willingness to help

An additional 15 individuals ranked 30 charity projects from low to high according to their willingness to help. Among them, we selected the top four (range of ranking: 11.20 ± 10.185–13.33 ± 9.186), middle two (range of ranking: 15.60 ± 7.763; 15.87 ± 8.314), and last four (range of ranking: 17.73 ± 7.750–18.87 ± 8.052) charity projects to measure willingness to help. Thus, we chose 10 charity projects from 30 projects.

##### 3.1.2.3 Honesty measurement

**Figure 1:**
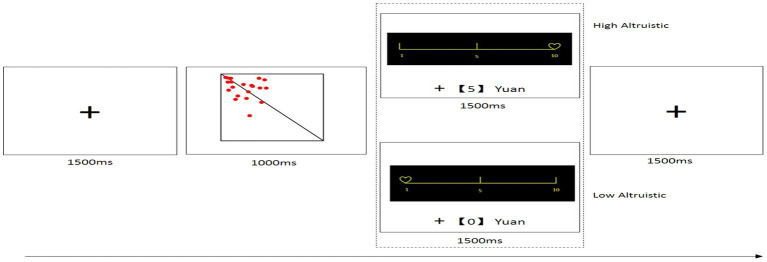
Image example and trial procedure.

To measure honesty, a visual perception task (Gino et al., 2010; Gino and Ariely, 2012; Zhou et al., 2018) was used in this study. In this task, participants are shown a square with dots on either side of a line. The participants then report whether there are more dots on the right side of the line, more on the left side, or the dots are equally divided between both sides. The task begins with a practice phase of 15 trials, and the official experiment contains 50 trials (clear conditions: 17 trials with more than 2/3 of the dots on the left side, eight trials with more than 2/3 of the dots on the right side; unclear condition: 25 trials fewer than 2/3 of the dots on either side; Figure 1).

For each selection of “more red dots on the right side,” participants would receive 5 yuan (high reward). For each selection of “more red dots on the left side,” participants would receive 0.5 yuan (low reward). If participants failed to provide a response in 1,000 ms, they would receive 0 yuan.

Considering that the reward for choosing “more dots on the right side” was 10 times more than that for choosing “more dots on the left side,” Zhou et al. (2018) noted that, in the clear condition in which there were more dots on the left side, individuals who selected the high-reward button lied when facing a conflict between honesty and high reward, indicating a choice to give up their confident self-image to pursue high self-interest. Contrastingly, individuals who selected the low-reward button in this condition were honest in the event of a conflict between honest behavior and high reward.

#### 3.1.3 Procedure

This experiment included four stages. In the first stage, the participants engaged in a practice experiment for the perception task to familiarize themselves with its key operations. In the second stage, they were asked to read about the 10 charity projects (e.g., the “Masks with Love” charity project). They then ranked these projects based on their willingness to help. In the third stage, the participants completed the fairness experience task, where they were randomly assigned the recipient role from one of three groups: negative unfairness, positive unfairness, or fairness.

In the final stage, participants (as recipients) took part in the high/low altruistic perception task. They were informed that they could enter the formal part of this task with the money allocated to them in the previous round, an opportunity not extended to the allocator. This stage comprised two groups: the high altruistic group and the low altruistic group. In the high altruistic group, the initial screen informed the participants that the beneficiary of the bonus obtained in this stage was the charity project that had been ranked highest in terms of their willingness to help. A fixation point (“+”) then appeared on the screen for 1,500 ms. Subsequently, a square with a red dot appeared at the center of the screen, and the participant had to key in their judgment within 1,000 ms. Pressing the F key indicated “more dots on the left,” while the J key indicated “more dots on the right.” The icon of the charity project with the highest willingness to help rating and the accrued amount was then displayed for 1,500 ms. Pressing the F key provided a “+ [0] yuan” message and pressing the J key provided a “+ [5] yuan” message. If no key was pressed, a “Reaction too slow, + [0] yuan” message was displayed. In the low altruistic group, the initial screen informed the participant that the bonus beneficiary in this stage was the charity project that had been ranked lowest in terms of their willingness to help. The feedback interface displayed the icon of the charity project with the lowest willingness to help. Other than these two aspects, the low altruistic group followed the same setup as the high altruistic group. Trials were presented to each group randomly, with each group participating in 50 trials, resulting in a total of 100 trials (Figure 1). Participants saw a high or low charity project before making a choice.

### 3.2 Results

After eliminating data from 18 participants whose error rate exceeded three standard deviations from the mean (Zhou et al., 2018) for points on the right, 93.68% of valid data remained for analysis. This data comprised 89 participants from the negative unfairness group, 88 from the fairness group, and 90 from the positive unfairness group. Thus, a total of 267 participants (160 females, 107 males; mean age = 20.288, SD = 1.267, age range = 18–24) were evaluated.

First, a repeated measures analysis of variance (ANOVA) was conducted using a 3 (within-subjects variable: type of fairness experience [fairness, negative unfairness, positive unfairness]) × 2 (within-subjects variable: type of conflict [conflict, non-conflict]) × 2 (within-subjects variable: type of motivation [high altruistic, low altruistic]) design. The results showed significant main effects for both types of conflict (*F*(1, 264) = 760.090, *p* < 0.001, *η*_p_^2^ = 0.742) and motivation (*F*(1, 264) = 65.006, *p* = 0.000, *η*_p_^2^ = 0.198). The non-conflict group’s key press accuracy (M ± SD = 0.671 ± 0.011) was significantly higher than that of the conflict group (M ± SD = 0.267 ± 0.019), and the high altruistic group’s key press accuracy (M ± SD = 0.531 ± 0.015) was significantly higher than that of the low altruistic group (M ± SD = 0.407 ± 0.016). A significant interaction was observed between the type of conflict and type of motivation (*F*(1, 264) = 64.295, *p* = 0.000, *η*_p_^2^ = 0.196). A simple effects analysis showed that in the conflict condition, the high altruistic group’s accuracy (M = 0.294, SD = 0.022) significantly surpassed that of the low altruistic group (M = 0.240, SD = 0.020, *p* = 0.007). In the non-conflict condition, the high altruistic group’s accuracy (M = 0.768, SD = 0.012) was significantly greater than that of the low altruistic group (M = 0.573, SD = 0.015, *p* = 0.000). Under the high altruistic condition, the non-conflict group’s accuracy (M = 0.768, SD = 0.012) was significantly higher than that of the conflict group (M = 0.294, SD = 0.022, *p* = 0.000). Under the low altruistic condition, the non-conflict group’s accuracy (M = 0.573, SD = 0.015) was significantly higher than that of the conflict group (M = 0.240, SD = 0.020, *p* = 0.000). However, the main effect of the three types of experiences of fairness was not significant (*F*(2, 264) = 0.214, *p* = 0.807, *η*_p_^2^ = 0.002), and other interaction effects were also insignificant.

Subsequently, the reaction time for the correct key press of the high-reward response served as the dependent variable in the 3 (type of fairness experience) × 2 (type of conflict) × 2 (type of motivation) repeated measures ANOVA. After excluding the data of participants who did not press the high-reward response key, the participant pool comprised 41 individuals from the negative unfairness group, 36 from the fairness group, and 41 from the positive unfairness group, totaling 118 participants. The results revealed a significant main effect for the type of conflict (*F*(1, 115) = 27.373, *p* = 0.000, *η*_p_^2^ = 0.192). The non-conflict type had a significantly longer response time for an accurate key press (M ± SD = 421.474 ± 11.815) compared to the conflict type (M ± SD = 381.590 ± 11.863). A significant interaction was found between the type of motivation and type of fairness experience (*F*(2, 115) = 3.504, *p* = 0.033, *η*_p_^2^ = 0.057). A simple effect analysis demonstrated that, under high altruistic conditions, the accurate key-press response time of the negative unfairness group (M = 366.947, SD = 19.586) was significantly shorter than that of the fairness group (M = 424.430, SD = 20.902, *p* = 0.047). Interestingly, under positively unfair conditions, the accurate key-press response time of the high altruistic group (M = 390.869, SD = 19.586) was significantly shorter than that of the low altruistic group (M = 432.868, SD = 22.372, *p* = 0.022). However, the main effects of type of motivation (*F*(1, 115) = 1.945, *p* = 0.166, *η*_p_^2^ = 0.017) and type of fairness experience (*F*(2, 115) = 0.873, *p* = 0.420, *η*_p_^2^ = 0.015), and all other interactions, was insignificant.

Therefore, the likelihood of individuals lying in an altruistic context is significantly influenced by the type of conflict and the high and low altruistic motives, while the response time is significantly influenced by the interaction of conflict type, high and low altruistic motives, and the type of fairness experience. First, under non-conflict conditions, individuals were more likely to lie, albeit with a slower response time, than they were in conflict conditions. Second, when it came to a charity project with a higher willingness to help, participants exhibited a greater inclination to lie compared to a project with a lower willingness to help. Third, in the context of a charity project with a higher willingness to help, the response time of participants who had experienced negative unfairness was shorter than that of participants who had encountered a fairness. Lastly, for participants who had experienced positive unfairness, the response time to lie for a charity project with a higher willingness to help was shorter than the response time to lie for a project with a lower willingness to help.

## 4 Experiment 2: ERP study of experiences of fairness and honest behaviors

### 4.1 Materials and methods

#### 4.1.1 Participants

We recruited 74 adult college students from a university in East China; two were excluded due to excessive artifacts, leaving 72 (14 females, 58 males; age: M = 19.81, SD = 1.370, age range = 18–23) in the final analysis. The participants were all right-handed, and none had visual impairments. Prior to the start of the experiment, all participants signed an informed consent form. This study received ethical approval from the Soochow University’s ethics committee, and participants were assigned a participant number at random to ensure anonymity. At the end of the experiment, all participants were compensated, each receiving 70 Yuan.

#### 4.1.2 Design

The allocation of experiences of fairness and the measure of willingness to help were identical to that of Experiment 1.

##### 4.1.2.1 Honesty measurement

This experiment used a coin-guess task to measure the spontaneous dishonesty of the participants (Hu et al., 2015; Cui et al., 2018; Lu et al., 2019). In this task, the participants could win a bonus by reporting whether their prediction of the coin toss was correct. Two scenarios were involved in this game: participants either had the opportunity to lie or they did not. In the first scenario, participants were presented with the phrase “random random” on the interface used for predicting the face of the coin and were asked to mentally predict the outcome. Given this arrangement, researchers were unable to evaluate if participants were lying. Conversely, in the second scenario, participants saw the phrase “number pattern” on the interface, and they were required to report their prediction outcomes by pressing a key. In this context, researchers were able to accurately determine whether the participants were lying.

### 4.2 Procedure

This experiment comprised four stages. During the initial stage, participants completed a practice round of the coin-guess task, familiarizing themselves with the two distinct scenarios. E-Prime software was used during this practice stage to display experimental stimuli and record behavioral data.

In the second stage, the participants were provided with materials related to 10 different charity projects (e.g., “Masks with Love”) along with their descriptions. The participants were then asked to rank these projects by their willingness to help.

The third stage involved randomly assigning participants to a type of fairness experience, wherein they were designated an identity from one of three groups: negative unfairness, fairness, or positive unfairness.

**Figure 2:**
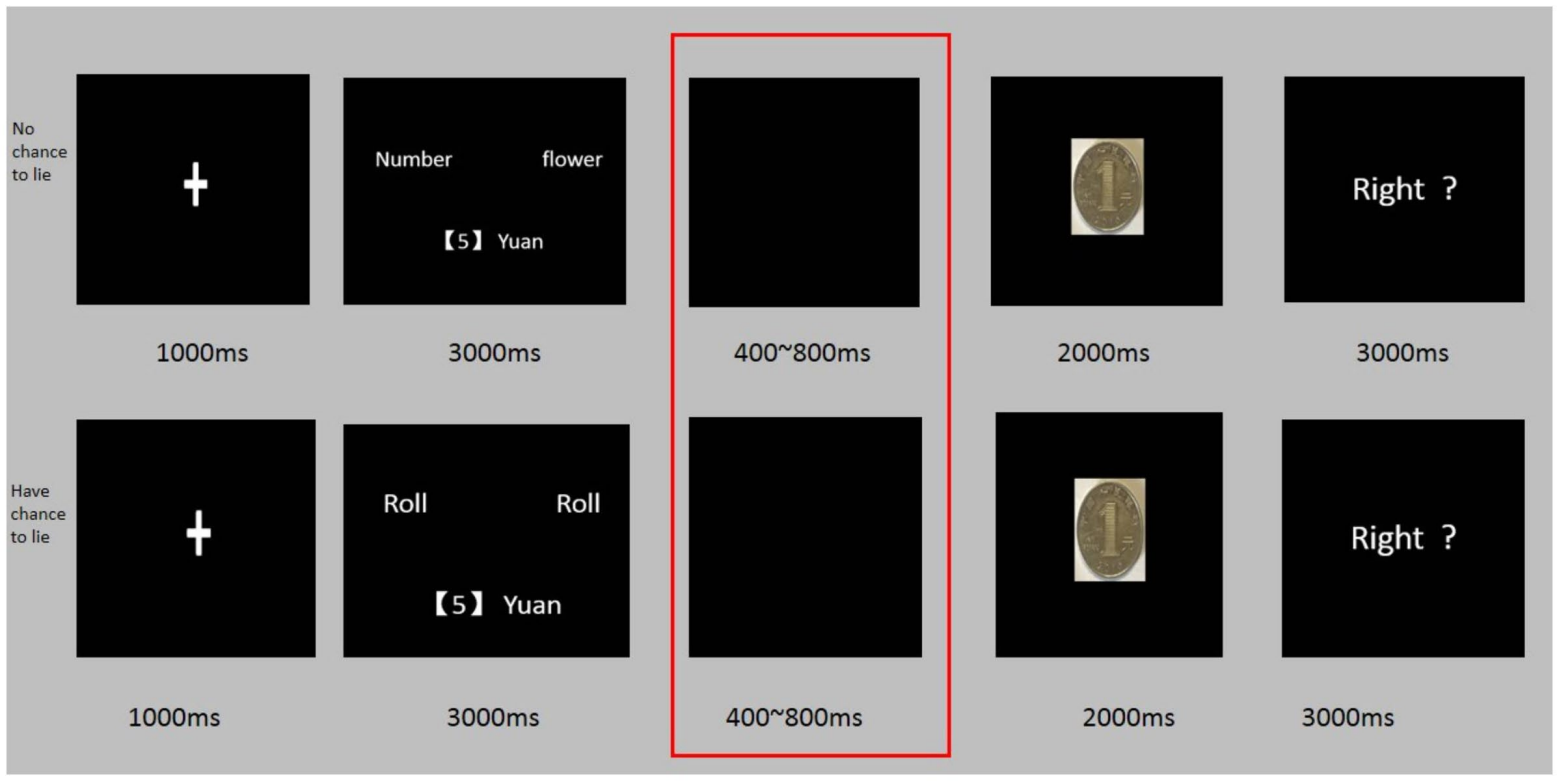
Trial procedurals of two rules in coin-guess task.

In the fourth stage, participants proceeded to the formal experiment of the coin-guess task, which was divided into two sections. Before each section, a screen would notify the participants about the beneficiary of the winnings from the section, either the highest-ranked (high-altruistic condition) or the lowest-ranked (low-altruistic condition) charity project. The sequence of these two sections was evenly distributed among the participants. Each section of the coin-guess task comprised two types of trials: those in which there was an opportunity to lie, and those in which there was not. Therefore, Experiment 2 comprised four within-subject conditions: high-altruistic condition with no opportunity to lie, high-altruistic condition with an opportunity to lie, low-altruistic condition with no opportunity to lie, and low-altruistic condition with an opportunity to lie. Figure 2 further illustrates this process. During the trials with no opportunity to lie, a fixation point (“+”) was displayed for 1,000 ms, followed by numbers and patterns appearing on the left and right sides of the screen. Participants had 3,000 ms to report their prediction by pressing either the F key for numbers or the J key for patterns. A blank screen was then displayed for 400–800 ms before revealing the result of the coin toss for 2,000 ms. The screen subsequently displayed the question, “Did you guess right?,” and participants indicated the accuracy of their prediction (pressing Y for correct or N for incorrect). The reward for this condition was then shown. In the trials with no opportunity to lie, the setup remained largely similar to that of the other trial, except for the prediction interface. In the no-opportunity-to-lie trials, numbers and patterns were presented, while in the opportunity-to-lie trials, random elements were displayed, and participants only needed to press the R key to proceed, without the need to choose numbers or patterns. Each condition included 54 trials, totaling 216 trials. Specifically, there were 54 trials with no opportunity to lie and 54 trials with opportunity to lie in high or low altruistic conditions, respectively. After completing all the trials in each condition, participants were given a 2-min rest. Demographic information was collected after the experiment.

### 4.3 Data recording and analysis

An EEG recording and analysis system with 64 channels, manufactured by Brain Products, Germany, was used for this experiment. In alignment with the International 10–20 System, the grounding point was established at the GND point. Electrodes were positioned 1.5 cm away from the left and right eyes to record the horizontal electrooculogram, while others were situated approximately 1 cm above and below the left eye to record the vertical electrooculogram. The Fz point served as the reference electrode throughout the experiment. Impedance between the scalp and the electrode was maintained below 5 kΩ, the band-pass filter ranged from 0.01–30 Hz, and the sampling frequency was set at 500 Hz. Blink artifacts were eliminated using ICA, and artifact signals ±80 μV were excluded.

The outcomes of the coin toss were recorded from 200 ms before the screen display to 1,000 ms after the screen display, with measurement at 200 ms before the screen display used as the baseline. The screen displaying the coin toss results was chosen for analysis as it was within this display that participants first ascertained the accuracy of their prediction and made the decision about whether to lie. The analyzed ERP components included the N2 and P3 components. Based on existing research findings and the overall average topographic map of current data (Hu et al., 2015; Cui et al., 2018), the N2 component (250–350 ms) primarily spans the frontal and fronto-central scalp regions, with the selected electrodes being F3, F4, Fz, FC3, FC4, and FCz. The P3 component (300–450 ms) is predominantly distributed in the centro-parietal and parietal scalp regions, with the selected electrodes being CP3, CP4, CPz, P3, P4, and Pz.

The IBM SPSS 18 was used to conduct statistical analysis. For each component, the selected electrode points within the specified time window were analyzed using a 3 (type of fairness experience: fair, positive unfairness, negative unfairness) × 2 (type of trial: with opportunity to lie, no opportunity to lie) × 2 (type of motivation: high altruistic, low altruistic) repeated measures ANOVA for the peak and average amplitudes under the varying conditions.

### 4.4 Results

#### 4.4.1 Behaviors: accuracies and reaction times

The 3 × 2 × 2 repeated measures ANOVA was targeting the participants’ self-reported accuracy rates. The analysis revealed a significant main effect for the type of trial (*F*(1, 69) = 14.354, *p* = 0.000, *η*_p_^2^ = 0.172). The reported accuracy rate was higher (M = 0.5700, SD = 0.1542) when participants had an opportunity to lie compared to when they did not (M = 0.5019, SD = 0.0868). Similarly, the type of motivation exhibited a significant main effect (*F*(1, 69) = 8.957, *p* = 0.004, *η*_p_^2^ = 0.115), where the reported accuracy rate was higher under the high altruistic condition (M = 0.5700, SD = 0.1303) compared to the low altruistic condition (M = 0.5172, SD = 0.1263). However, the type of fairness experience did not present a significant main effect (*F*(2, 69) = 0.077, *p* = 0.926, *η*_p_^2^ = 0.002). Furthermore, no significant interaction was observed between the type of trial and type of fairness experience (*F*(2, 69) = 0.066, *p* = 0.939, *η*_p_^2^ = 0.022), type of motivation and type of fairness experience (*F*(2, 69) = 0.163, *p* = 0.850, *η*_p_^2^ = 0.005), type of trial and type of motivation (*F*(1, 69) = 0.341, *p* = 0.561, *η*_p_^2^ = 0.005), or any three-way interaction (*F*(2, 69) = 1.726, *p* = 0.186, *η*_p_^2^ = 0.048).

These findings demonstrate that, during the completion of the coin-guess task, the participants’ judgments of the coin’s sides were significantly impacted by both trial type and motivation type. More specifically, the reported accuracy in the with-opportunity-to-lie condition was significantly greater than that in the no-opportunity-to-lie condition. Additionally, the reported accuracy in the high altruistic condition was significantly higher than that in the low altruistic condition.

The same 3 × 2 × 2 repeated measures ANOVA was performed to analyze participants’ self-reported reaction times. The analysis revealed a significant main effect for trial type (*F*(1, 66) = 6.256, *p* = 0.014, *η*_p_^2^ = 0.087), where the reaction time for correctly reported responses in the with-opportunity-to-lie condition (M = 500.125, SD = 24.282) was significantly longer than that in the no-opportunity-to-lie condition (M = 462.809.125, SD = 18.703). Conversely, the main effects for fairness experience type (*F*(2, 66) = 2.092, *p* = 0.132, *η*_p_^2^ = 0.060) and motivation type (*F*(1, 66) = 0.975, *p* = 0.327, *η*_p_^2^ = 0.015) were not significant. Furthermore, no significant interactions were observed between trial type and fairness experience type (*F*(2, 66) = 0.523, *p* = 0.595, *η*_p_^2^ = 0.016), motivation type and fairness experience type (*F*(2, 66) = 1.301, *p* = 0.279, *η*_p_^2^ = 0.038), trial type and motivation type (*F*(1, 69) = 0.213, *p* = 0.646, *η*_p_^2^ = 0.003), or any three-way interaction (*F*(2, 66) = 0.471, *p* = 0.626, *η*_p_^2^ = 0.014).

These results indicate that participants’ reaction time was significantly influenced by the type of trial; that is, the time taken for the participants to report correctly under the with-opportunity-to-lie condition was significantly longer than it was under the no-opportunity-to-lie condition.

#### 4.4.2 Event-related potentials

**Figure 3:**
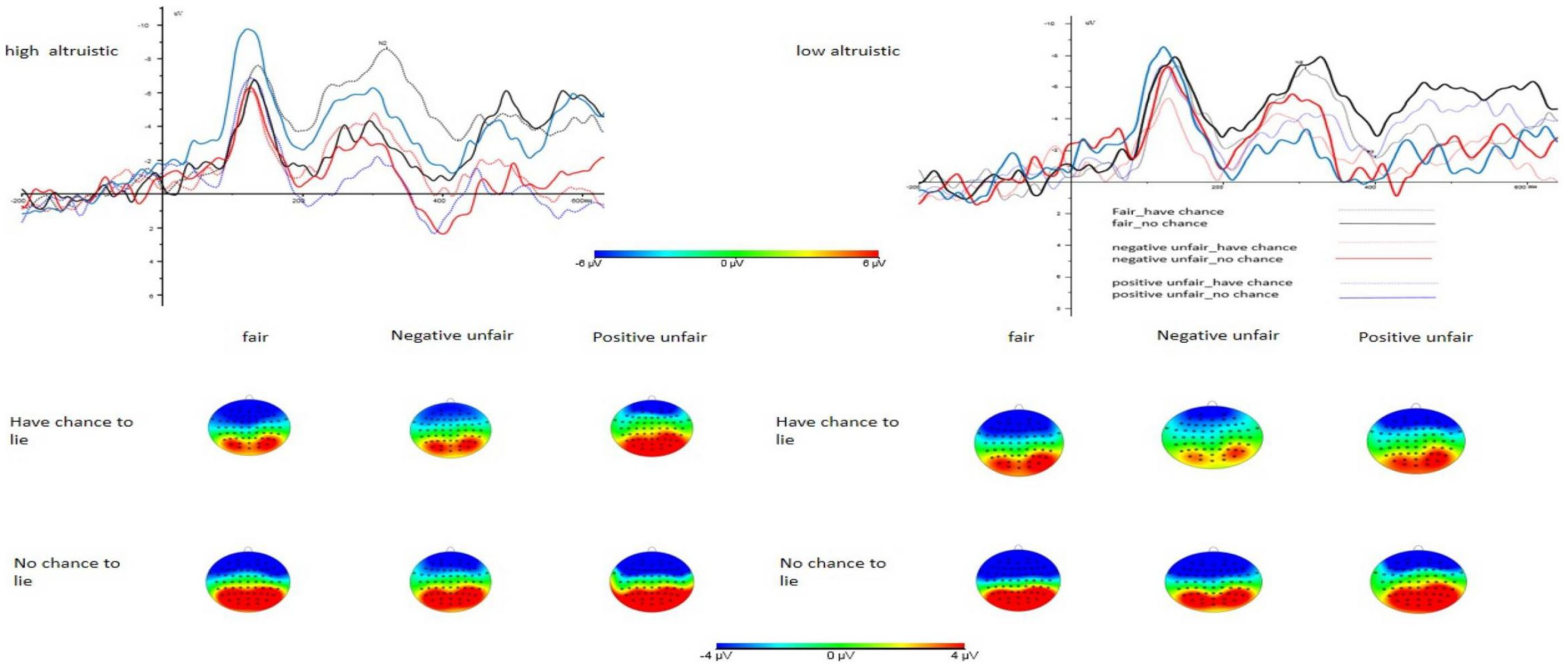
Waveforms and topography of N2 components (Fz electrode points) under high and low altruistic conditions.

The same 3 × 2 × 2 repeated measures ANOVA was performed for the amplitude of the N2 component. None of the three main effects proved to be significant (type of fairness experience: *F*(2, 69) = 0.573, *p* = 0.566, *η*_p_^2^ = 0.016; type of trial: *F*(1, 69) = 0.020, *p* = 0.888, *η*_p_^2^ = 0.000; type of motivation: *F*(1, 69) = 1.195, *p* = 0.278, *η*_p_^2^ = 0.017). Likewise, the interactions between type of trial and type of fairness experience (*F*(2, 69) = 1.146, *p* = 0.324, *η*_p_^2^ = 0.022), type of motivation and type of fairness experience (*F*(2, 69) = 0.855, *p* = 0.430, *η*_p_^2^ = 0.005), and type of trial and type of motivation (*F*(1, 69) = 0.344, *p* = 0.599, *η*_p_^2^ = 0.005) were insignificant. However, the three-way interaction effect between type of fairness experience, type of trial, and type of motivation was significant (*F*(2, 69) = 4.077, *p* = 0.021, *η*_p_^2^ = 0.106). A simple effect analysis revealed that under the with-opportunity-to-lie and high altruistic condition, the N2 component amplitude was larger for the fairness group (M = −9.798, SD = 1.155) than it was for the positive unfairness group (M = −5.683, SD = 1.130, *p* = 0.013). Under the positive unfairness and high altruistic condition, the no-opportunity-to-lie condition (M = −9.613, SD = 1.822) induced a larger N2 component amplitude than it did in the with-opportunity-to-lie condition (M = −5.683, SD = 1.130) (*p* = 0.017). In the positive unfairness and with-opportunity-to-lie condition, the low altruistic condition (M = −7.995, SD = 1.217) induced a larger N2 component amplitude than the high altruistic condition (M = −5.683, SD = 1.130, *p* = 0.040) (see Figure 3).

These findings suggest that the amplitude of the N2 component is significantly influenced by the interaction between trial type, motivation type, and fairness experience type. Specifically, under the with-opportunity-to-lie and high altruistic condition, participants exposed to fair distribution induced a larger negative wave than those who experienced positive unfair distribution. Under the positive unfairness and high altruistic condition, the with-opportunity-to-lie condition induced a smaller negative wave than the no-opportunity-to-lie condition. Lastly, under the positive unfairness and with-opportunity-to-lie condition, the lowest-rated charitable project in terms of willingness to help induced a larger negative wave than the highest-rated project.

**Figure 4:**
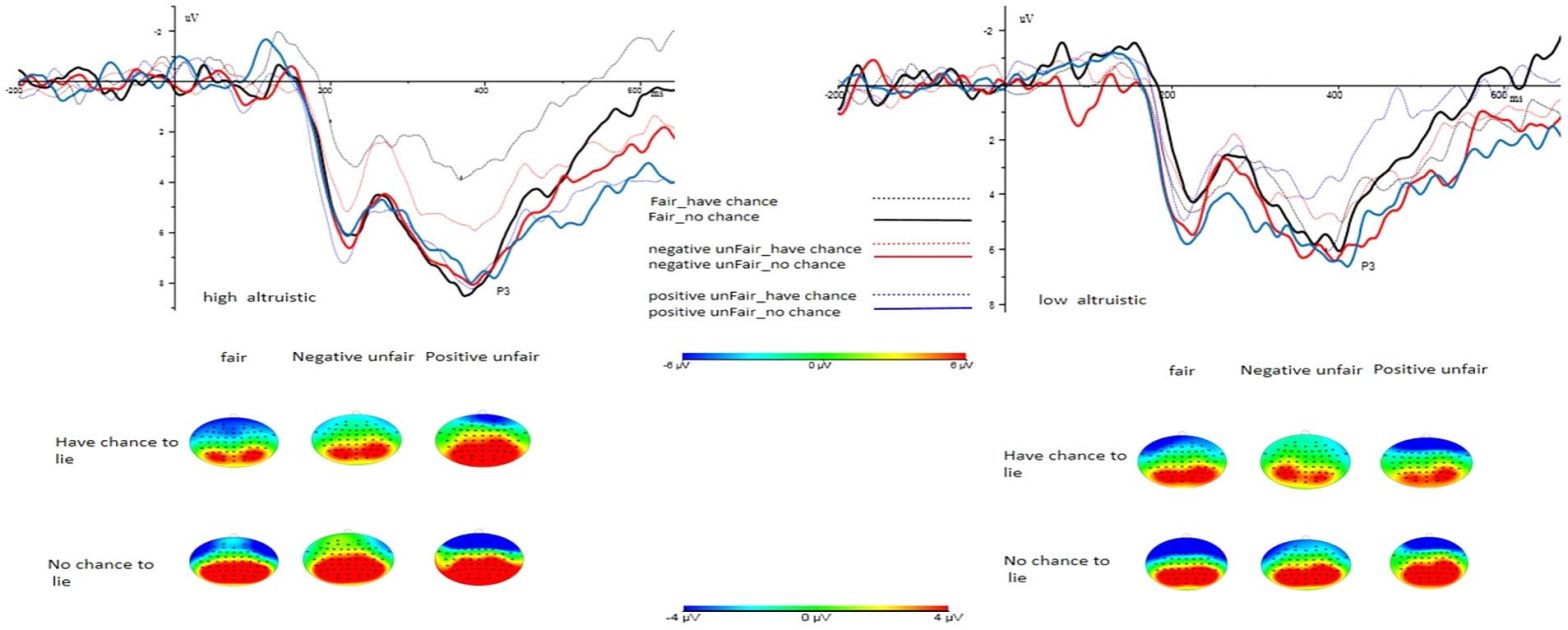
P3 component (P3 component electrode point) waveforms and topography under high and low altruistic conditions.

The same 3 × 2 × 2 repeated measures ANOVA was performed for the amplitude of the P3 component. A significant main effect was observed for trial type (*F*(1, 69) = 7.218, *p* = 0.009, *η*_p_^2^ = 0.095), with the P3 component amplitude for trials with the opportunity to lie (M = 7.624, SD = 0.693) being significantly lower than those with no opportunity to lie (M = 9.074, SD = 0.756). The other two main effects, relating to fairness experience type (*F*(2, 69) = 0.342, *p* = 0.712, *η*_p_^2^ = 0.010) and motivation type (*F*(1, 69) = 1.045, *p* = 0.310, *η*_p_^2^ = 0.015), were found to be insignificant. Similarly, the interactions between trial type and fairness experience type (*F*(2, 69) = 0.254., *p* = 0.776, *η*_p_^2^ = 0.007), motivation type and fairness experience type (*F*(2, 69) = 1.606, *p* = 0.208, *η*_p_^2^ = 0.044), and trial type and motivation type (*F*(1, 69) = 0.000., *p* = 0.988, *η*_p_^2^ = 0.000) were not significant. However, a significant three-way interaction was observed among fairness experience type, trial type, and motivation type (*F*(2, 69) = 4.324, *p* = 0.017, *η*_p_^2^ = 0.111). The simple effect analysis results indicated that under the with-opportunity-to-lie and high altruistic condition, the positive unfairness group (M = 10.230, SD = 1.503) triggered a larger positive wave than the fairness group (M = 5.740, SD = 1.535, *p* = 0.040). Furthermore, under the fairness and high altruistic condition, the with-opportunity-to-lie condition (M = 5.740, SD = 1.535) induced a smaller positive wave than the no-opportunity-to-lie condition (M = 9.303, SD = 1.449, *p* = 0.003). Under the advantageous unfairness and low altruistic condition, the with-opportunity-to-lie condition (M = 7.033, SD = 1.098) induced a smaller positive wave than the no-opportunity-to-lie condition (M = 9.358, SD = 1.483, *p* = 0.070). Additionally, under the positive unfairness and opportunity-to-lie condition, the high altruistic condition (M = 10.230, SD = 1.503) produced a larger positive wave than the low altruistic condition (M = 7.033, SD = 1.098) (*p* = 0.004). Lastly, under the fairness and with-opportunity-to-lie condition, the low altruistic condition (M = 7.912, SD = 1.121) triggered a larger positive wave than the high altruistic condition (M = 5.740, SD = 1.535, *p* = 0.054) (see Figure 4).

The amplitude of the P3 component was discernibly influenced by the main effects of the trial and the interaction between trial type, motivation type, and fairness experience type. Specifically, participants induced a smaller P3 amplitude under the with-opportunity-to-lie condition compared to the no-opportunity-to-lie condition. Under the with-opportunity-to-lie and high altruistic condition, participants who experienced fairness elicited a smaller positive wave than those who experienced positive unfairness. In the fairness and high altruistic condition, the with-opportunity-to-lie condition induced a smaller positive wave than the no-opportunity-to-lie condition. Under the positive unfairness and with-opportunity-to-lie condition, donations to assist the lowest-ranking charity project induced a smaller positive wave than those to assist the highest-ranking charity project.

## 5 Discussion

The main purpose of this study was to explore the predictive impact of experiences of fairness on honest behavior, where the key comparison was between fairness, positive unfairness, and negative unfairness. Through these two experiments, we analyzed the likelihood of people behaving dishonestly to advance the interests of others based on whether the other party was perceived to have high or low levels of altruism. Our study provides boundary conditions for the effects of different levels of altruistic motivation on individuals’ honest or dishonest behavior. We found that individuals who had positive unfairness experiences felt less conflict and exerted less mental effort when lying for a high altruistic counterpart compared to a less altruistic counterpart. This is evident in their shorter reaction times, contradicting *Hypothesis 1*.

Positive experiences of unfairness evoked smaller N2 and larger P3 wave amplitudes when individuals were engaged in dishonest behavior to help a high altruism counterpart, compared with those who had fair experiences. This finding partly supports *Hypothesis 2* and provides neurological evidence for the existing findings of Gino and Pierce (2009), who found that only wealthy graders would behave dishonestly to help poor solvers. Similarly, Xu et al. (2020) found that the late LPP/P300 elicited by fair offers was more positive than moderately negative unfair offers. However, there are two differences between our findings and those of Xu et al. (2020). First, our findings focused on the comparison between fairness and positive unfairness, while Xu et al. (2020) focused on the comparison between fairness and negative unfairness. Second, we only obtained these results when participants helped their high altruism counterparts, while the findings of Xu et al. (2020) appeared regardless of whether the charity was deemed to have deserved assistance.

Cui et al. (2018) noted that, when moral conflict was less intense (smaller N2 wave amplitude), less mental effort was required to resolve it (P3 wave amplitude). This indicates that, in settings with opportunities to lie, individuals with positive unfairness experiences suffer less moral conflict (inducing smaller N2 amplitude) and expend less mental effort and fewer cognitive resources to resolve this conflict (inducing larger P3 amplitude) than those with fair experiences when they lie for a high altruistic counterpart. However, regarding reaction time to dishonest behavior, we found that, when faced with a highly altruistic counterpart, those with negative unfairness experiences behaved dishonestly with shorter reaction times than those who had experienced fairness. Existing research states that behavioral reaction times typically reflect the fact that the behavior is influenced by a decisional conflict, and the less psychological conflict individuals feel when making behavioral decisions, the faster they react (Evans and Rand, 2019). Thus, compared to those who had fair experiences, those with negative unfair experiences felt less moral conflict when lying for a high altruistic counterpart and thus reacted faster. This may be because people are more sensitive to negative events than positive ones (Baumeister et al., 2001; Klein and Epley, 2014; Kross et al., 2021), so people react more strongly when being treated unfairly than when being treated fairly (Leib et al., 2019).

Furthermore, our findings indicate that individuals are more likely to engage in dishonest behavior when dealing with a highly altruistic counterpart compared to a less altruistic counterpart. This finding aligns with previous studies. Specifically, a behavioral study conducted by Lu et al. (2019) revealed that participants were more willing to lie if it served their own interests rather than the interests of their less altruistic counterparts. However, Lu et al. (2019) did not observe any distinctions between high altruistic counterparts and less altruistic or self-interested counterparts.

### 5.1 Theoretical and practical implications

First, the recent study adds to the controversial hypothesis implications of cross-norm inhibition effect. Cross-norm inhibition effect has been supported (Keizer et al., 2008). Our findings provide more empirical support to refute the conventional cross-norm inhibition effect. As a result, more specific restrictive circumstances may be necessary for the cross-norm inhibition effect, which warrants further consideration.

Second, this study also adds to the body of research on the connection between fairness and ethical behavior. We found that only experiences of fairness, as opposed to experiences of unfairness, have a stable impact on honest conduct. Although earlier research examined the link between many aspects of fairness and moral action, the circumstances and brain mechanisms in which this relationship occurred were frequently disregarded. As a result, the present study’s findings are novel in that they close this research gap.

Third, this paper’s findings will advance understanding of the variables affecting ethical decision-making. Numerous environmental factors that may affect people’s decision-making for present-day and ethical behaviors have been the content of earlier studies. Studies already published have assessed moral conduct from a fairness perspective (Houser et al., 2012; Galeotti et al., 2017). Our findings add to this body of research by demonstrating that people’s dishonest behavior may be affected by unfairness or fairness, which can be considered as specific contextual element.

Fourth, this paper provide implications of these findings for real-world situations. From the perspective of fairness experience, our findings provide a new path for the moral decision-making of college students. Positive fairness experience is a research topic worthy of the attention of scholars and administrators in university and college.

### 5.2 Limitations and future directions

First, this study focused on the impact of fairness experiences on self-serving dishonesty. However, in a future study, we will extend from an altruistic scenario to a self-serving one. Additionally, we will explore the differences in the influence of fair experiences on moral behavior between altruistic and self-serving scenarios.

Second, this study elucidated the brain responses or neural mechanisms by which fairness influences altruistic dishonesty, but did not explain more specifically the mechanisms involved in the formation of this. Thus, future research could prepare new experiments to examine this mechanism.

Third, a limitation of this paper is the relatively small sample size. Additionally, it focuses only on altruistic dishonesty. Future studies should provide more details about the participants with potential impact in confusing self-serving and altruistic dishonesty and enlarge the sample size of the participants.

## Data availability statement

The original contributions presented in the study are included in the article/supplementary materials, further inquiries can be directed to the corresponding author.

## Ethics statement

The studies involving humans were approved by Soochow University’s ethics committee. The studies were conducted in accordance with the local legislation and institutional requirements. The participants provided their written informed consent to participate in this study. Written informed consent was obtained from the individual(s) for the publication of any potentially identifiable images or data included in this article.

## Author contributions

CZ: Conceptualization, Data curation, Investigation, Methodology, Visualization, Writing – original draft, Writing – review & editing. MY: Funding acquisition, Software, Writing – review & editing. JW: Conceptualization, Funding acquisition, Investigation, Supervision, Writing – review & editing.

## References

[ref1] Adams J. S. (1965). “Inequity in social exchange” in Advances in Experimental Social Psychology. ed. Berkowitz L., vol. 2 (New York: Academic Press), 267–299.

[ref9001] Baumeister R. F. Bratslavsky E. Finkenauer C. Vohs K. D. (2001). Bad is stronger than good. Rev. Gen. Psychol. 5, 323–370. doi: 10.1037/1089-2680.5.4.323

[ref2] Cui F. Wu S. Wu H. Wang C. Jiao C. Luo Y. (2018). Altruistic and self-serving goals modulate behavioral and neural responses in deception. Soc. Cogn. Affect. Neurosci. 13, 63–71. doi: 10.1093/scan/nsx138 29149322 PMC5793826

[ref3] Evans A. M. Rand D. G. (2019). Cooperation and decision time. Curr. Opin. Psychol. 26, 67–71. doi: 10.1016/j.copsyc.2018.05.007 29879640

[ref4] Galeotti F. Kline R. Orsini R. (2017). When foul play seems fair: exploring the link between just deserts and honesty. J. Econ. Behav. Organ. 142, 451–467. doi: 10.1016/j.jebo.2017.08.007

[ref5] Gino F. Ariely D. (2012). The dark side of creativity: original thinkers can be more dishonest. J. Pers. Soc. Psychol. 102, 445–459. doi: 10.1037/a0026406 22121888

[ref6] Gino F. Norton M. I. Ariely D. (2010). The counterfeit self: the deceptive costs of faking it. Psychol. Sci. 21, 712–720. doi: 10.1177/0956797610366545 20483851

[ref7] Gino F. Pierce L. (2009). Dishonesty in the name of equity. Psychol. Sci. 20, 1153–1160. doi: 10.1111/J.1467-9280.2009.02421.x 19674386

[ref8] Gino F. Pierce L. (2010). Lying to level the playing field: why people may dishonestly help or hurt others to create equity. J. Bus. Ethics 95, 89–103. doi: 10.1007/s10551-011-0792-2

[ref9] Houser D. Vetter S. Winter J. (2012). Fairness and cheating. Eur. Econ. Rev. 56, 1645–1655. doi: 10.1016/j.euroecorev.2012.08.001

[ref10] Hu X. Q. Hegeman D. Landry E. Rosenfeld J. P. (2015). Increasing the number of irrelevant stimuli increases ability to detect countermeasures to the P300-based complex trial protocol for concealed information detection. Psychophysiology 49, 85–95. doi: 10.1111/j.1469-8986.2011.01286.x, PMID: 22091554

[ref11] Keizer K. Lindenberg S. Steg L. (2008). The spreading of disorder. Science 322, 1681–1685. doi: 10.1126/science.1161405 19023045

[ref12] Klein N. Epley N. (2014). The topography of generosity: asymmetric evaluations of prosocial actions. J. Exp. Psychol. Gen. 143, 2366–2379. doi: 10.1037/xge0000025 25313952

[ref9002] Kross E. Verduyn P. Sheppes G. Costello C. K. Jonides J. Ybarra O. (2021). Social media and well-being: Pitfalls, progress, and next steps. Trends Cogn. Sci. 25, 55–66. doi: 10.1016/j.tics.2020.10.005 33187873

[ref13] Leib M. Moran S. Shalvi S. (2019). Dishonest helping and harming after (un)fair treatment. Judgm. Decis. Mak. 14, 423–439. doi: 10.1017/s1930297500006112

[ref14] Lu J. Liao C. Guan Q. Luo Y. Cui F. (2019). Deceptive behaviors under the altruistic and egoistic motivations: an ERP investigation. J. Psychol. Sci. 42, 905–912. doi: 10.16719/j.cnki.1671-6981.20190420

[ref15] Miraghaie A. M. Pouretemad H. Villa A. E. Mazaheri M. A. Khosrowabadi R. Lintas A. (2022). Electrophysiological markers of fairness and selfishness revealed by a combination of dictator and ultimatum games. Front. Syst. Neurosci. 16:765720. doi: 10.3389/fnsys.2022.765720 35615426 PMC9124946

[ref16] Xu Q. Yang S. Huang Q. Chen S. Li P. (2020). A sense of unfairness reduces charitable giving to a third-party: evidence from behavioral and electrophysiological data. Neuropsychologia 142:107443. doi: 10.1016/j.neuropsychologia.2020.107443, PMID: 32240667

[ref17] Zhou X. Q. Yan L. L. Wang Z. Hu X. K. Xu Y. J. (2018). The effect of watching eyes on dishonest behavior. Psycho. Explor. 38, 333–338.

